# Impressions of American Rheumatology and Recent Advances in "Rheumocrinology"
*Introduction to a discussion at the Bath Clinical Society, November, 1949.


**Published:** 1950-07

**Authors:** G. D. Kersley


### IMPRESSIONS OF AMERICAN RHEUMATOLOGY
### AND
### RECENT ADVANCES IN "RHEUMOCRINOLOGY" *

BY

G. D. KERSLEY, T.D., M.D., M.A., F.R.C.P.

---

Apart from the recent discoveries in connection with the endocrine glands and rheumatic disease, the most striking feature at the International Congress held in New York in June 1949 was the truly American burst of enthusiasm for research into, and treatment of this most neglected group of diseases. In some places endowment of research into rheumatism is so great that the main problem is to know how the annual income can be spent—the complete reverse of the situation in this country! Attendance at meetings, even after lunch in a real heat wave, was almost one hundred per cent., though proceedings of one kind or another usually started at 7.30 a.m. and finished after midnight.

In some section or other of the Congress nearly all aspects of rheumatic disease were discussed. The morbid histology of the muscles in rheumatoid arthritis was considered to be nonspecific and probably biochemical in origin, but occurred seldom except in rheumatoid muscles. The Americans still disagree with the British over the question of whether ankylosing spondylitis is or is not rheumatoid arthritis of the spine. The selection of the cases and pre- and post-operative treatment of cup arthroplasty of the hip appeared to be even more important than the technique of the operation. Excision of the heads of the metatarsals is proving useful in rheumatoid deformity of the feet. The importance of heredity in rheumatoid arthritis and spondylitis was stressed and also the way the hereditary hyperuricaemia of gouty families was controlled by the gonads. The effect of the thyroid, pituitary and gonads on the ageing of

* Introduction to a discussion at the Bath Clinical Society, November, 1949.

bone in osteoarthritis was also agreed. It was, however, on the role of the adrenal cortex and pituitary that interest mainly centred; and we had an opportunity of seeing a number of cases treated both with cortisone and A.C.T.H.—yet during the whole of the meeting I did not hear Selye's name mentioned once.

### Rheumatism as a Disease of Adaptation

For many years we have been exercised by the problem of why such divergent factors as heredity, shock, worry, infection, fatigue, exposure and allergy have all appeared to produce the same end-picture. Now an explanation appears to be forthcoming. In 1936 Selye described the "Alarm Reaction and General Adaptation Syndrome". He found that in animals a large number of noxious stimuli—extreme heat or cold, extreme fatigue, crushing of muscle, poisoning with such drugs as colchicine—all produced the same result:

1. A stage of shock.
2. Recovery and increased resistance to the stimulus; and
3. If the stimulus was continued, death in exhaustion.

There were atrophy of the thymus, lymphoid tissues and spleen, depletion of the adrenal cortical lipoid, renal changes and ulceration of the bowel. The change was identical, whatever the noxious stimulus.

Hench drew attention to the reversibility of the rheumatoid process, especially in pregnancy and jaundice, and encouraged the biochemists to renew their efforts at isolation and synthesis of the adrenal steroids. In 1942 Kendal isolated 17–hydroxy–11–dehydrocorticosterone, later named cortisone, and in 1946 succeeded in its partial synthesis. In 1948, Hench, Slocumb and Polley investigated its therapeutic potentialities.

In 1946 Selye produced arthritis in rats by removing both adrenal glands and one kidney, and then keeping them alive on D.O.C.A. (desoxycorticosterone) and salt. He followed this up in 1949 by the production of arthritis in rats with formaldehyde injection in the region of the joints and aggravating this with D.O.C.A. or controlling it with cortisone or A.C.T.H.

When A.C.T.H., one of the hormones produced by stimulation of the anterior part of the pituitary gland, reacts on the adrenal cortex, three groups of steroid hormones are produced:

1. "Sex" hormones—androgens, oestrogens and progesterone-like substances.

2. D.O.C.A.-like substances, sometimes called the electrolytic or mineralo-corticoids, which are used in the treatment of Addison's disease and are mainly concerned with electrolytic water balance; and

3. The 11–oxysteroids, sometimes described as the carbohydrate– or glyco-corticoids, which affect the deposition of glycogen in the liver and the production of carbohydrates from protein. It is to this group that compound E or cortisone—17–hydroxy–11–dehydrocorticosterone—belongs.

Group 2 tends to have an adverse effect and group 3 a beneficial effect on rheumatic disease. The effect of the latter is measured most easily in the laboratory by the depression of the eosinophil level in the circulating blood and an increase in the uric acid in the urine, usually expressed as an increase in the uric acid/creatinine ratio.

*Cortisone*, given by injection in a dose of 100 to 200 mg. daily, produces a dramatic temporary remission in rheumatoid arthritis, ankylosing spondylitis and rheumatic fever. It is also effective in generalized lupus erythematosis, scleroderma, erythema nodosum; and in asthma, ulcerative colitis, mental disease, and possibly nephrosclerosis and hyperpiesis. In rheumatoid disease, within twenty-four hours there is diminution of spasm, pain and swelling and increase in range of movement of the affected joints. There is a sense of euphoria and increase in appetite and weight. In about a week there is usually a 90 per cent. recovery of all except incapacity due to structural changes in bone or firm fibrous changes in the capsules. The sedimentation rate and blood count improve greatly: an increase of as many as one million red cells has been observed in this period. Within two days of cessation of treatment a relapse is, however, to be expected, and by the end of a week about 80 per cent. of the gain is often again lost.

*A.C.T.H.* has a therapeutic effect almost identical with that of cortisone in rheumatic disease. It stimulates the adrenal cortex, except in the rare cases when the latter is diseased, to pour into the circulation more of the cortisone-type than of D.O.C.A.-like steroids. It is, however, still more quickly absorbed and destroyed, and is usually given by intra-muscular injections in doses of about 30 mg. three times a day.

*Danger, Difficulties and Significance.* At first cortisone and A.C.T.H. appear a panacea for many of man's ills, but there are many difficulties. With regard to production, cortisone is only synthesized in minute quantities by a very laborious process from large quantities of ox bile. A.C.T.H. is with equally great difficulty and expense obtained from animals' pituitary. The possibility of synthesis of A.C.T.H. has, however, become a little nearer since the recent work of Li and Morris (1950). The production of anti-hormones, especially with A.C.T.H., are a very real difficulty. And, in addition, with both A.C.T.H. and cortisone, there is a risk of adrenal failure if their administration is long continued and then suddenly stopped.

Toxic effects during cortisone administration are infrequent; inhibition of scar tissue formation and breaking down of wounds has been reported. With A.C.T.H. in particular, acne, hirsuties and amenorrhoea are to be looked for, but they usually right themselves rapidly on cessation of treatment. Contamination with pitressin may produce rise in blood pressure, faintness and vomiting.

From the above remarks it must be agreed that neither cortisone nor A.C.T.H. should at present be looked upon as a treatment. Their discovery does, however, open up an enormous field for research and helps to explain much that was previously not understood about the possible causation of the rheumatic diseases. Professor Williams has put forward a very reasonable conception : of a group of diseases of adaptation, where there is a constitutional weakness somewhere in the chemistry of the body, probably in one of the fundamental enzyme systems, so that when subjected to stress, there is a breakdown at this level. When excess of substrate is available, the chemical reaction can continue, and the supply of abnormal amounts of substrate or the prevention of its destruction may be the therapeutic answer. The need for the use of cortisone in a dose ten times that calculated for the requirements of the body and the clinical remission of rheumatoid arthritis with jaundice by reduction of steroid destruction by the liver are both explicable by this fascinating theory. It also explains how heredity and such factors as shock, fatigue, allergy and infection can all play their part in production of the rheumatic state.

*Ascorbic Acid and D.O.C.A.* In 1949 Lewin and Wassen stated that they had produced dramatic remissions in rheumatoid arthritis by the simultaneous injection of 5 mg. D.O.C.A. intramuscularly and 0.5 to 1.0 gr. ascorbic acid intravenously. Brownless in part confirmed this work in animals. He found that formalin-produced arthritis was worsened by D.O.C.A., but improved by D.O.C.A. and ascorbic acid even after removal of the adrenals. He also found some improvement with ascorbic acid alone, provided that the adrenals were intact. Le Vey and Loxton claimed good results with D.O.C.A. and ascorbic acid, even when the ascorbic acid was given intramuscularly in the same dosage (0.5 to 1.0 gr.). They also were able to localize the improvement to one limb by giving the injections into that limb and restricting the venous outflow with a cuff, and therefore suggest that any effect of the injections was peripheral and not by stimulation of the adrenals. These authors also claim that equally good results were obtained in stiffness following trauma in which " rheumatism " played no part.

Most observers (especially those with research and clinical experience of the rheumatic diseases) have failed to produce these dramatic results with ascorbic acid and D.O.C.A. or other steroids, although occasional improvement is seen, usually where there has been a strong psychological build-up. A drop in circulating eosinophils and increase in uric acid excretion, which invariably occur with cortisone or A.C.T.H., are not usual with ascorbic acid and D.O.C.A. (Kersley, *et als*). The papers in which brilliant results have been claimed show no control series: and any observer of rheumatic cases knows, not only how naturally variable the disease is, but how psychology influences the condition temporarily in a dramatic way. In any case, where improvement following the use of D.O.C.A. and ascorbic acid is claimed, the relief of pain and stiffness, usually commencing in five minutes to two hours, is not, as a rule, continued for more than one day; there is no evidence of lasting benefit, and no observer has recorded systemic benefit, gain in weight, drop in sedimentation rate, or improvement of the anaemia. It is felt that, while the use of ascorbic acid and other oxidizing-reducing agents given in conjunction with certain steroids merits investigation, the widespread therapeutic application of D.O.C.A. and ascorbic acid is not at present justified.

### Summary

The use of endocrine preparations is not as yet at a stage where it can be considered as routine therapy. Though recent discoveries have given us a better understanding of the rheumatic diseases, opened up new lines of research and brightened the hope for the future, they have in no way altered our older lines of therapy—the general care of the individual patient, physically and mentally, the elimination of the excitant factors and local treatment of the affected joints. There is at the moment no justification for the widespread use of ascorbic acid and D.O.C.A. in clinical practice.

### REFERENCES

1. Brownless, G. (1950).—*Lancet*, **1**, 157.
2. Copeman, W. S. C., *et al* (1950).—*B.M.J.*, **1**, 1006.
3. Fletcher, E., *et al* (1950).—*Lancet*, **1**, 94.
4. Hart, V. L. (1949).—*Lancet*, **2**, 1203.
5. Hench, P. S., *et al* (1949).—*Proc. Mayo Clin.*, **24**, 8, 181.
6. Ib. (1949).—*Ann. Rheum. Dis.*, **8**, 97.
7. Kellgren, J. H. (1949).—*Lancet*, **2**, 1108.
8. Kersley, G. D., *et al* (1950).—*Lancet*, **1**, 703.
9. Le Vey and Loxton (1950).—*Lancet*, **1**, 209.
10. Lewin, F., and Wassen, E. (1949).—*Lancet*, **2**, 993.
11. Li quoted by Sir R. Robinson (1949).—*Nature*, **164**, 1029.
12. Morris, P., and Morris, C. J. (1950).—*Lancet*, **1**, 117.
13. Selye, H. (1936).—*Brit. J. Expt. Path.*, **17**, 234.
14. Ib. (1946).—*J. Clin. Endocrin.*, **6**, 117.
15. Ib. (1949).—*Brit. Med. J.*, **2**, 1129.
16. Williams, R. J., *et al* (1950).—*Lancet*, **1**, 287.

